# Refinement of a mouse cardiovascular model: Development, application and dissemination

**DOI:** 10.12688/f1000research.14456.1

**Published:** 2018-05-15

**Authors:** Kirk A. Taylor, Michael Emerson

**Affiliations:** 1 Platelet Biology Group, National Heart and Lung Institute, Imperial College London, London, SW7 2AZ, UK

**Keywords:** endothelium, mortality, mouse, pharmacology, platelet, reduction refinement, thrombosis

## Abstract

European and UK legislation requires all animal procedures to be conducted with consideration to reduction, refinement and replacement. In this review, 3Rs developments are discussed in the field of platelet biology and thromboembolism. Platelet research requires the use of animal models, and mice are widely used in the field. When working *in vitro*, conventional light transmission techniques have been scaled down allowing reduction in animal numbers. *In vivo*, vascular injury models are widely used and work is ongoing to develop *ex vivo* approaches that use fewer animals. Thromboembolic mortality models, which inflict considerable pain and suffering, have also been used widely. A published and characterised refinement of this mortality model allows real-time monitoring of radiolabelled platelets under general anaesthesia and reduces both the severity level and the numbers of mice used in a typical experiment. This technique is more sensitive than the mortality approach and has opened up new avenues of research, which would not have been feasible by using death as an end-point. To drive uptake of real-time monitoring, a more simplistic approach has been developed involving micro-sampling and cell counting. Thromboembolic mortality models should therefore be considered obsolete due to the emergence of 3Rs models with improved scientific outcomes and that can be implemented relatively easily.

Research highlights **Scientific benefits:**

- Acquisition of pharmacodynamics dose-responses increases sensitivity
- Platelet function is assessed directly

**3Rs benefits:**

- Procedures are conducted at a lower severity level avoiding pain and suffering.
- Animal numbers are reduced by 85%

**Practical benefits:**

- Simplistic alternative micro-sampling approaches are available that require only standard laboratory equipment and minimal training

**Current applications:**

- Investigating cardiovascular risk associated with exposure to airborne pollution and HIV/antiretroviral therapy

**Future applications:**

- Broadly within the fields of anti-thrombotic therapy and platelet physiology/pharmacology
- Potential for further reduction in the field by developing flow cytometric approaches for multiple analyses of diluted micro-samples

## Cardiovascular diseases and platelets

Cardiovascular diseases including myocardial infarction (MI) and stroke are the major cause of death in Western society. Cardiovascular risk is heightened by the unavoidable risk factor of age, but is also driven by avoidable, or potentially avoidable, factors such as smoking, poor diet and airborne pollution ^1^. Cardiovascular disease is also increasingly important to clinicians and scientists in other fields. For example, cardiovascular events are the major cause of death in people living with HIV who are virally supressed by antiretroviral therapy ^2^.

When investigating mechanisms of cardiovascular disease, platelets are of obvious interest. Platelets are circulating blood elements with central roles in haemostasis and thrombosis and are a major therapeutic target in the treatment of arterial thrombotic events such as MI. Aspirin and clopidogrel inhibit platelet activation and are widely prescribed in patients following MI, although their efficacy remains suboptimal and new therapeutic approaches are required.

When activated, platelets undergo a series of signalling processes involving calcium release and the expression of activation markers, including P-selectin, on the extracellular surface. Platelets also release a range of inflammatory mediators from intracellular granules, including chemokines such as PF4 (Platelet Factor 4) and CCL5 (chemokine (C-C motif) ligand 5). Platelets therefore generate a range of signals that drive processes such as adhesion, aggregation and inflammation. Human platelets are easily isolated from blood and their function may be assessed through a range of established assays. The gold-standard functional assay remains light transmission aggregometry, which measures increases of light transmission through initially opaque platelet suspensions as platelets aggregate ^3^.

## *In vitro* and *ex vivo* analysis of platelet function

Platelets are anucleate and it is not possible to alter protein expression using traditional *in vitro* strategies, such as siRNA knockdown. Therefore, it is often necessary to employ mouse models where protein expression of the precursor cell, the megakaryocyte, can be altered. A significant reduction achievement concerning *in vitro* mouse platelet aggregometry has been the development of 96-well plate light transmission approaches ^4^, which use much smaller plasma volumes than conventional cuvette approaches. By reducing the standard plasma volume from 450 to 90 μl, a reduction in mouse use of 80% is possible when switching to a 96-well format. In addition, running multiple samples in parallel in a 96-well plate, rather than consecutive cuvette analyses, can reduce sample variability and thus reduce numbers of animals required per experiment.

Flow cytometry is widely used in biomedical research and with an increasing number of available antibodies and markers that permit diverse analysis in very small samples of cells and fluids, this approach can dramatically increase the number of experimental parameters per experiment. Platelet researchers routinely monitor high affinity integrin α _IIb_β3, alpha and dense granule release as markers of platelet activation alongside assays to determine changes of Ca ^2+^ signalling and aggregation ^5– 8^. Emergent flow cytometers, such as the BD Accuri C6, enable agonists to be added during sample acquisition, allowing for simultaneous kinetic evaluation of platelet activation ^8^. A major advantage of this technology is that data can be obtained with diluted samples (i.e. 1:200), raising the possibility that micro-sampling could replace alternative blood collection techniques, such as retro-orbital sampling and cardiac puncture. Ongoing work in our laboratory is evaluating the potential of using flow cytometry to perform ratiometric analysis of agonist-evoked calcium signalling, platelet aggregation and protein studies using human samples. If successful, these techniques could be applied to mouse studies and reduce the overall number of animals required.

Reporting of numbers of animals used per experiment in the literature is highly variable and it is often not possible to determine whether the same animal has been used in multiple assays. Taking these limitations into account, we predict that measurement of platelet activation markers, aggregation response and calcium response to three platelet agonists using conventional assays would require 13 mice per experimental replicate. However, development of flow cytometric approaches discussed here could reduce this number by over 90%.

## Need for animal models

The involvement of multiple cell types and tissues in the etiology of cardiovascular diseases creates a strong necessity for animal studies, and more than 63,000 animal procedures involving mice were reported in the Cardiovascular, Blood and Lymphatic field by the UK Home Office in 2016 ( https://www.gov.uk/government/statistics/statistics-of-scientific-procedures-on-living-animals-great-britain-2016).

Within the field of thrombosis and platelets, vascular injury models involving endothelial damage via chemical, laser or mechanical trauma and subsequent thrombus formation are widely used ^9^. Although the technique is conducted terminally under general anaesthesia, the number of thrombi that can be recorded per animal is limited, since, for example, both carotid arteries cannot be ligated. In addition, the high variance of the data outputs mean that relatively high numbers of animals are required to statistically power studies. Efforts are currently underway to develop an *ex vivo* carotid artery model involving luminal cannulation of excised artery sections: https://www.nc3rs.org.uk/reducing-animal-use-thrombosis-research-ex-vivo-injury-model. In due course, it is hoped that this model will reduce mouse use by at least 50% since both carotid arteries can be used.

The vascular endothelium releases mediators such as nitric oxide (NO) and prostacyclin, which inhibit platelet function and are central to both haemostasis and the development of platelet-driven cardiovascular events ^10– 12^. Vascular injury models have proven to be problematic when used to assess the impact of endothelial mediators in platelet-driven thrombosis. In the NO field, ablation of endothelial NO synthase (eNOS) was shown to result in no phenotype ^13, 14^, an anti-thrombotic phenotype ^15, 16^ or a pro-thrombotic phenotype ^17^ by different groups using similar experimental approaches. Assessment of the role of endothelial mediators requires, rather than a vascular injury approach, a model in which platelets circulate freely in the context of a functional vascular endothelium, which can be manipulated pharmacologically or genetically ^18^.

## Animal models: Need for refinement

The need to model platelet function and thrombosis *in vivo* has led to the development of vascular injury models, which are conducted under general anaesthesia, but also to the use of models of thromboembolic mortality, which are conducted in conscious animals and, as the name suggests, use mortality as an end-point ^19^. Thromboembolic mortality models allow the assessment of platelet aggregation *in vivo* against the backdrop of a functional vascular endothelium. Mortality models involve the intravenous injection of thrombogenic agents such as collagen or thrombin with death or hind limb paralysis as an end-point. To quote from a 2003 publication ^20^: “ *Pulmonary embolism was induced in male Swiss-Webster mice by intravenous tail injection of a mixture of collagen and epinephrine. Doses were selected that resulted in death or at least 15 minutes of hind-limb paralysis in approximately 90% of control mice*”. The effects of genetic modification or drug action are measured by their ability to significantly change the proportions of mice killed or paralysed. This model has been used relatively recently to assess the roles of novel intracellular signalling pathways in platelets ^21^, endogenous compounds ^22^, platelet receptor antagonists ^23^, free radical scavengers ^24^, dietary compounds ^25^, endogenous nitric oxide ^10^ and a novel aspirin derivative ^26^ on thromboembolic mortality.

Induction of thromboembolism in mice undoubtedly inflicts considerable pain and suffering. Following injection, the mice adopt a hunched posture, become immobile and breathing becomes laboured ^19^. The mice remain in this state until they die or are assessed, by their inability to use their hind-limbs, to be paralysed. The rationale for conducting the procedure in conscious mice is that under anaesthetic, mortality is more difficult to induce ^19^ (presumably shock contributes to death and blood pressure is lower under anaesthesia) and paralysis cannot easily be determined. In addition to the severe impact upon the animals, this technique requires large numbers of subjects and studies have been published involving 20 or 40 animals per experimental group ^10, 27^, so that the total number in a publication can run into the hundreds.

## Refined model development

We have developed refined models for the assessment of platelet thromboembolism using monitoring of radiolabelled platelets in anaesthetised mice ^28, 29^. Our technique avoids the use of death as an end-point and instead measures the thromboembolic response in real-time by tracking radiolabelled platelets *via* externally placed scintillation probes ^28^. To achieve this, mice are terminally anaesthetised and bled by cardiac puncture, platelets are radiolabelled with a gamma emitter and then infused into a second terminally anaesthetised mouse. Thus, a model is created in which radiolabelled platelets circulate freely against the background of a fully functional vascular endothelium, and so retaining a key feature that has been used historically to justify the use of thromboembolic mortality models. The same range of thrombogenic substances that can be used to induce mortality, when given at lower doses, induce reversible and dose-dependent increases in platelet counts in a probe suspended over the pulmonary vascular bed. Thus, the whole time course of the platelet thromboembolic response may be recorded and quantified in a number of ways, including peak response and area under the curve ^28^. We were able to validate our model with the anti-platelet drug aspirin as a means of assessing its potential clinical relevance ^28^.

## Reduction

Despite the fact that real-time monitoring requires animals both for acquisition of platelets and monitoring, our approach allows not only refinement of procedures to a lower severity level, but has also reduced the numbers of animals required in a typical experiment. The extent of reduction is best exemplified when looking at work demonstrating the role of endogenous NO in inhibiting platelet activation *in vivo*. This was first demonstrated in mice in 1998 when administration of a NO synthase inhibitor was shown to increase thromboembolic mortality in mice ^10^. This paper required the use of 200 mice to demonstrate the antithrombotic activity of endogenous NO and additional studies (including *in vivo* studies in other species) to link mortality studies with a platelet-mediated effect. A comparable study using refined real-time platelet monitoring involved only 30 mice with all procedures performed under general anaesthesia (classified as non-recovery severity level under current Home Office legislation). Thus the refined model led to an 85% reduction in mouse use in a typical experiment ^18^.

## Scientific application of refined model

Real-time platelet monitoring produces dynamic and quantifiable read-outs and provides dose-dependent platelet accumulation responses. This contrasts with mortality studies which simply measure the occurrence, or lack of, an event. The refined model should therefore provide greater sensitivity since we are able to detect shifts in pharmacodynamic dose-responses, reflecting either enhanced or reduced platelet activation. We suggest that recent work in the field of cardiovascular risk in the context of airborne pollution demonstrates this ^30, 31^. It has been known for many years that exposure to pollution leads to increased cardiovascular morbidity and mortality and, in particular, exposure to particulate pollution increases myocardial infarction, a platelet-driven cardiovascular event ^32^. Due to their physicochemical properties, combustion-derived nanoparticles such as diesel exhaust particles (DEP) have been strongly implicated in driving cardiovascular risk ^33^. DEP have been shown to induce inflammation ^34^ and due to their nanoparticulate nature are hypothesised to translocate across the pulmonary epithelial barrier into the blood ^35^, which would bring them into direct contact with circulating platelets. Having shown that DEP can interact physically with platelets and induce their aggregation in *in vitro* human studies ^30^, it was necessary to proceed to *in vivo* studies to translate this finding to a more relevant whole organism setting. It was shown that both introduction of DEP to circulating blood to mimic translocation ^30^ and tracheal instillation of DEP to mimic inhalation ^31^ resulted in a significantly enhanced platelet thromboembolic response at doses of DEP that reflected human exposure levels. These studies contributed to the now more widely accepted thinking that DEP exposure may enhance cardiovascular risk in the human population and provided a potential mechanism. We suggest that this type of work, which highlights relatively subtle events that require sensitive models, would not have been possible in mortality models, certainly not at doses of DEP that were relevant to human exposure levels.

## Dissemination and uptake

3Rs benefits of animals arise not from their development, which creates potential, but from their subsequent employment in studies where non-refined models would have been used in the absence of refined alternatives. Examples from our own group have been discussed above and there are other outcomes in the fields of nanotoxicology ^36^, nutritional biochemistry ^37^ and calcium signalling ^38^. We have also collaborated with other groups in the fields of cyclooxygenase pharmacology ^12^, integrin linked kinase ^8^ and sulforophane (an isothiocyanate with potential antithrombotic activity; unpublished study, authors: Gillespie, Holloway, Becker, Rauzi, Vital, Shreveport, Taylor, Stokes, Emerson and Gavins).

The ultimate aim of 3Rs research is broad uptake of emerging 3Rs technologies by the scientific community and a consequential reduction in the use of non-refined procedures. Quantification of continued use of thromboembolic mortality models is difficult since their use is often obscured through lack of inclusion in abstracts and use of vague terminology. PubMed searches of [thromboembolism + mouse] and [thrombosis + mortality + mouse] and [platelet + mortality + mouse], followed by a manual search to identify publications revealed more than 50 papers where thromboembolic mortality was used in 2013. A review of these papers found that all used mortality and/or paralysis as end-points, the duration of the experiments varied from 5 minutes to 96 hours, and experimental groups varied from 10 to 30 mice, with 10 to 360 mice used in total in each paper. Similar numbers of papers appear in the 5 years preceding 2013.

More recently, in 2016 ^39^, we sampled 9 peer review articles using models of thromboembolic mortality and found between 3 and 40 animals per experimental group, with some studies not reporting animal use, the duration of the period in which animals were observed following induction of thromboembolism varied from 5 min to 5 days. Anaesthesia of any sort was only used in one study. Unfortunately, thromboembolic mortality models are therefore still used despite the availability of an alternative refined model. Potential reasons for continued use of thromboembolic mortality models are: Lack of awareness of alternatives, animal welfare not a primary consideration, inability to work with radioisotopes, lack of expertise and preference for collaboration rather than establishment of new technologies

Increasing awareness of the 3Rs and refined models is an ongoing endeavour and we continue to work collaboratively. In addition, we set out to develop refined methods to study platelet thromboembolism *in vivo* that could be more widely adopted since they could be conducted with minimal training, non-specialised equipment and at low cost. We have now developed a model that allows thromboembolism to be assessed by measuring the fall in circulating platelets that occurs during thromboembolism in blood microsamples ^39^. Microsamples are taken from the tail vein of anaesthetised mice and repeated sampling allows for counts to be measured before and during the thromboembolic response in an individual animal. This technique allows thromboembolism to be assessed without the need for specialised equipment and without radioisotopes.

## Conclusions

In conclusion, platelet function can now be assessed *in vitro* and *in vivo* using 3Rs approaches that reduce the severity level and also the numbers of animals used in procedures. Thromboembolic mortality approaches should now be considered obsolete, since even where the gold-standard real-time monitoring technique cannot be employed, more simplistic assays can be used without the need for radioactive material or complex procedures.

## Data availability

No data are associated with this article.
