# Supplementary material for: Refinement of a mouse cardiovascular model: Development, application and dissemination
Source: F1000Res. 2018 May 15;7:593. [Version 1] doi: 10.12688/f1000research.14456.1 (PMC5974574; doi:10.12688/f1000research.14456.1)
Supplement: Peer review history

# Peer review: Reviewer response for version 1

**DOI:** 10.5256/f1000research.15737.r34024

**Published:** 2018-05-25

**Authors:** Alan G.S. Harper

**Affiliations:** 1 Institute for Science and Technology in Medicine, Keele University, Keele, UK

Mice are widely used in the platelet research field - this is largely due to this species’ accessibility to genetic modification and intravital microscopy. However due to their small blood volume and size and the variable responses to surgical treatments, these experiments often require the use of significant number of mice to reproduce the experiments to get statistically significant data. Therefore there is a key need to identify 3Rs strategies to help platelet researchers minimise the harm done to mice used in this field.

 In this report, the authors provide an interesting and insightful review into the 3R’s approaches that have been applied to both *ex vivo* and *in vivo* studies of platelet function in mice. Taylor and Emerson provide excellent examples of how 3Rs approaches are being used and developed in their laboratory to help refine and reduce the use of mice in platelet research. Of the different approaches, the refinement of the thromboembolic model provides a particularly valuable case study into how 3Rs approaches can not only improve animal welfare but also improve the quality of the scientific data obtained.  

 Whilst the authors discuss barriers to the dissemination of their technique, it would have been interesting to have seen more detailed description of these barriers, and a consideration of potential ways they could be overcome to further increase the 3Rs impact of this work. For example, a more detailed assessment of the training, financial and infrastructure needs required to adopt this technique and how this lead to the development of the cell counting model, would have provided an interesting insight into how to improve the impact of 3Rs interventions.

 The discussion of the uses of the thromboembolic and vascular injury models identifies some key differences between these models, however the review seems to more consider the relative merits of these two techniques as approaches to studying thrombosis generally, rather than perhaps more clearly describe their complementary roles in examining thrombosis and haemostasis – with the vascular injury models better replicating normal haemostatic reactions in the absence of a complete endothelial lining rather than true arterial thrombosis, whilst the thromboembolic model better reflects the processes of venous thromboembolism where coagulation is artificially triggered inside the intact venous system. However the key trigger of the acute cardiovascular events mentioned by the authors in their introduction is atherosclerotic plaque rupture.  The review therefore would be benefit from a brief mention of animal models of plaque rupture to give a more complete picture of the field.  Current murine atherosclerosis models are hampered by the lack of spontaneous plaque rupture with models using requiring a mechanical trigger to elicit the full pathological response ^1^, therefore future 3Rs work may also be needed on common non-mouse models in rabbits, pigs and non-human primates ^2^.

 For *ex vivo* studies, the authors provide some interesting case studies on how adoption of improved technology can pave the way to reduce blood volumes required to perform these studies, and as such reduce the number of mice utilised.  The authors show that these have the potential to have a major impact in reducing the number of animals. However this section does not appear to fit with the title of the review – therefore the review is brief and omits some promising 3Rs *ex vivo* approaches. The most important omission is of studies aiming to replace the use of mice in platelet research by attempting to recreate the physical and chemical environments of the circulation *ex vivo *using microfluidic and tissue engineering techniques ^3^. ^^These are beginning to provide a viable alternative to current animal models, and should also be considered as a potential future target of 3Rs interventions in platelet research.

**Does the review provide an accurate account of the 3Rs landscape in that field / across fields?**

 Partly

Is the review written in accessible language?

Yes

Are all factual statements correct and adequately supported by citations?

Yes

Are the conclusions drawn appropriate in the context of the current research literature?

Yes

Is the topic of the review discussed comprehensively in the context of the current literature?

Yes

Reviewer Expertise:

Platelet physiology, calcium signalling, vascular tissue engineering

I confirm that I have read this submission and believe that I have an appropriate level of expertise to confirm that it is of an acceptable scientific standard.

## References

[1] : Complementary roles of platelets and coagulation in thrombus formation on plaques acutely ruptured by targeted ultrasound treatment: a novel intravital model. *J Thromb Haemost*.2009;7(1) : 10.1111/j.1538-7836.2008.03186.x 152-61 10.1111/j.1538-7836.2008.03186.x 18983512

[2] : Animal models of atherosclerosis. *Eur J Pharmacol*.2017;816: 10.1016/j.ejphar.2017.05.010 3-13 10.1016/j.ejphar.2017.05.010 28483459

[3] : Primary Human Lung Alveolus-on-a-chip Model of Intravascular Thrombosis for Assessment of Therapeutics. *Clin Pharmacol Ther*.2018;103(2) : 10.1002/cpt.742 332-340 10.1002/cpt.742 28516446PMC5693794

# Peer review: Reviewer response for version 1

**DOI:** 10.5256/f1000research.15737.r34026

**Published:** 2018-05-23

**Authors:** David J. Grieve

**Affiliations:** 1 Centre for Experimental Medicine, Queen's University Belfast, Belfast, UK

This is an interesting and important article describing development of an improved experimental model of thromboembolism with clear potential to both replace and refine existing models. Whilst the manuscript is generally well written and presented, I do think that it could be significantly improved by revising structure and increasing 3Rs emphasis. Specific comments below.

 The title of the article does not accurately reflect its content. It is too general and should be specifically focussed on thromboembolism with regard to development of an improved experimental model. The stated scientific benefits in the research highlights box could also be made more specifically and understandable on a stand-alone basis.

 The article would benefit from inclusion of one of two figures to complement the text and make the article more engaging. For example, a summary of current experimental models used in platelet research with approximation of numbers of animals used would be helpful and it would also seem appropriate to include a schematic representation of the new model highlighting potential benefits.

 Need for animals models: the flow of this section could be improved. I do not think that the final paragraph really fits as currently written. More specific detail should also be provided in relation to the provided NC3Rs link e.g. research group etc.

 With specific regard to thromboembolism models, some discussion in relation to where these procedures are currently performed (e.g. are they permitted in the UK?) and comparison of scientific end-points between the new and existing models would be informative in order to highlight relevance and applicability.

 Dissemination and uptake: this section is quite weak at present and could be improved by including discussion of e.g. current barriers to uptake and potential approaches to encourage wider implementation. At present, discussion is largely limited to the authors own collaborators and in line with NC3Rs policy more needs to be done (which I am sure that it is) to promote wide uptake of new experimental models across the field.

 Abstract – specify light transmission aggregometry techniques.

**Does the review provide an accurate account of the 3Rs landscape in that field / across fields?**

 Yes

Is the review written in accessible language?

Yes

Are all factual statements correct and adequately supported by citations?

Yes

Are the conclusions drawn appropriate in the context of the current research literature?

Yes

Is the topic of the review discussed comprehensively in the context of the current literature?

Yes

Reviewer Expertise:

Referee suggested by the NC3Rs for their scientific expertise and experience in assessing 3Rs impact. Additional expertise: cardiovascular remodelling, heart failure, oxidative stress, endothelial proenitor cells, diabetes, experimental models, 3Rs.

I confirm that I have read this submission and believe that I have an appropriate level of expertise to confirm that it is of an acceptable scientific standard, however I have significant reservations, as outlined above.

# Peer review: Reviewer response for version 1

**DOI:** 10.5256/f1000research.15737.r34028

**Published:** 2018-05-22

**Authors:** Sarah Jones

**Affiliations:** 1 School of Healthcare Science, Manchester Metropolitan University, Manchester, UK

The availability of appropriate platelet function assays and thrombosis models is crucial to study mechanisms, which regulate the initiation and development of platelet rich thrombi, responsible for myocardial infarction and stroke. The study of specific proteins through altering expression levels in vitro is not possible in platelets since they do not have a nucleus. The use of genetically modified mice has therefore been crucial in furthering our understanding of platelet biology over the last two decades. European and UK legislations require that all animal procedures, are given suitable consideration to the 3Rs; reduction, refinement and replacement. In this review, Taylor and Emerson highlight some of the key developments in the platelet field, promoting the 3Rs principles, and discuss recent application of these developments. Critically, the review emphasises that impact on the 3Rs, can only be achieved through uptake of alternative technologies by the scientific community, which remains a significant challenge.

 The review gives a brief but comprehensive summary of recent techniques, which have had a significant impact on reducing animal numbers used in ex vivo platelet studies. These techniques allow for smaller blood volumes to yield meaningful functional data, reducing the number of animals sacrificed. Further on going research optimising flow cytometry techniques by the Emerson group, may ultimately lead to the acquisition of platelet function and signalling data, in blood samples obtained by micro-sampling. This could replace the need for blood collection methods such as cardiac puncture, which are performed under terminal anaesthesia.

 A brief mention is given to the existing vascular damage models of thrombosis. The authors should be cautious in this section, to be specific with the model that they are referring to and avoid over-generalization. While it is true that arterial thrombosis, mediated by ferric chloride (FeCl3 ) and measured by vascular occlusion can only asses one thrombi per animal and data is variable, this is not the case for laser injury, analysed by intravital microscopy. In this model, in excess of 20 thrombi can be analysed per animal and as few as 3 animals used in each experimental group ^1^^,^^2^.

 The review highlights the importance of endothelial derived platelet inhibitors in regulating thrombosis and haemostasis, and using nitric oxide as an example, indicates that vascular injury models have yielded contradicting results. While I am in agreement with this, I do not think that the approaches in the cited studies can be referred to as similar.  Differences in animal age, the mode and extent of vascular damage, and the type of vessel studied likely reflects the different results reported. Even studies using FeCl3 to injure the carotid artery vary significantly when FeCl3 is applied at different concentrations or for different lengths of time ^3^.

 As part of the discussion around measuring endothelial contributions to thrombosis, the authors state ‘Assessment of the role of endothelial mediators requires, rather than a vascular injury approach, a model in which platelets circulate freely in the context of a functional vascular endothelium, which can be manipulated pharmacologically or genetically.’ While this may be correct in a specific context, the appropriate model to investigate the role of endothelial derived mediators depends very much upon the research question. To investigate, the role of endothelial derived inhibitors on platelet aggregation in vivo, a model where platelets are circulating freely with a functional endothelium is appropriate. If however the contribution of the endothelium to arterial thrombosis or antithrombotic efficacy is being investigated, models, which encapsulate platelet adhesion, aggregation and thrombus stability in arterial conditions, following vascular damage, are more appropriate. It is also important to note that endothelial dysfunction is common in many diseases, which predispose to thrombosis, however currently there are no in vitro or in vivo models available to investigate the impact of this on thrombus generation.

 The review gives an excellent summary of the thromboembolism model developed by Emerson and colleagues, with well supported statistics of the significant impact on 3Rs that it has had so far. There is also good reference to application of the model in a variety of studies. There is good critique pertaining to limited uptake of the model by the scientific community, and potential reasons for this are discussed. The Emerson group have developed a further model, which is cost-effective, requires minimal training and non-specialised equipment, to try and address some of these issues and overcome the challenge of increased uptake. In summary, this review highlights important work carried out by the Emerson group, supported by the National Centre for the Replacement, Refinement and Reduction of Animals in Research, which has successfully developed, validated and applied refined real-time thromboembolisms models to replace the severe and archaic thromboembolism model that uses mortality or hind limb paralysis as an end-point.

**Does the review provide an accurate account of the 3Rs landscape in that field / across fields?**

 Yes

Is the review written in accessible language?

Yes

Are all factual statements correct and adequately supported by citations?

Yes

Are the conclusions drawn appropriate in the context of the current research literature?

Yes

Is the topic of the review discussed comprehensively in the context of the current literature?

Yes

Reviewer Expertise:

Platelets, endothelial cells, thrombosis

I confirm that I have read this submission and believe that I have an appropriate level of expertise to confirm that it is of an acceptable scientific standard.

## References

[1] : Real-time in vivo imaging of platelets, tissue factor and fibrin during arterial thrombus formation in the mouse. *Nat Med*.2002;8(10) : 10.1038/nm782 1175-81 10.1038/nm782 12244306

[2] : Integrin-linked kinase regulates the rate of platelet activation and is essential for the formation of stable thrombi. *J Thromb Haemost*.2014;12(8) : 10.1111/jth.12620 1342-52 10.1111/jth.12620 24888521

[3] : Towards a standardization of the murine ferric chloride-induced carotid arterial thrombosis model. *J Thromb Haemost*.2011;9(9) : 10.1111/j.1538-7836.2011.04287.x 1862-3 10.1111/j.1538-7836.2011.04287.x 21884567
